# Most British Surgeons Would Consider Using a Tissue-Engineered Anterior Cruciate Ligament: A Questionnaire Study

**DOI:** 10.1155/2012/303724

**Published:** 2012-02-26

**Authors:** Sarah Rathbone, Nicola Maffulli, Sarah H. Cartmell

**Affiliations:** ^1^School of Materials, Materials Science Centre, The University of Manchester, Manchester M1 7HS, UK; ^2^Centre for Sports and Exercise Medicine, Institute of Health Sciences Education, Barts and The London School of Medicine and Dentistry, Queen Mary University of London, Mile End Hospital, 275 Bancroft Road, London E1 4DG, England, UK

## Abstract

Donor site morbidity, poor graft site integration, and incorrect mechanical performance are all common problems associated with autografts for anterior cruciate ligament (ACL) reconstructions. A tissue-engineered (TE) ligament has the potential to overcome these problems. We produced an online questionnaire relating to tissue engineering of the ACL to obtain input from practising clinicians who currently manage these injuries. 300 British orthopaedic surgeons specialising in knee surgery and soft tissue injury were invited to participate. 86% of surgeons would consider using a TE ACL if it were an option, provided that it showed biological and mechanical success, if it significantly improved the patient satisfaction (63%) or shortened surgical time (62%). 76% felt that using a TE ACL would be more appropriate than a patellar tendon, hamstring, or quadriceps autograft. Overall, most surgeons would be prepared to use a TE ACL if it were an improvement over the current techniques.

## 1. Introduction

Some of the most frequently ruptured ligaments occur in the knee joint, often through sporting activities such as skiing, football, and basketball. Ninety percent of knee ligament injuries involve the anterior cruciate ligament (ACL) and medial collateral ligament (MCL) [1]. The MCL can self-heal, but the ACL is not able to do so from a combination of poor vascularisation and its intra-articular location. Therefore, alternative methods, including regenerative medicine, have focused heavily upon the ACL with the aim of producing a fully functional tissue *in vitro*. 

 The current gold standard procedure to reconstruct a torn ACL is surgical autografting. This involves using part of the patients own patellar, hamstring, or quadriceps tendon to replace the torn ACL [2]. However, this causes donor site morbidity [3–6] and is associated with pain and a recovery period for this region [5, 6]. Generally, 75–90% of patients have good or excellent long-term success rates from the current reconstruction techniques regarding functional stability and symptomatic relief upon return to normal activities, but many patients experience unsatisfactory results, which could be attributed to graft failure [7]. Some of these patients continue to endure pain, suffer from loss of motion secondary to the procedure, and their instability is not corrected [7]. Others suffer from degenerative joint disease such as osteoarthritis or experience reinjury [6]. Alternatively, allografts can be used; in this instance, the donor tendon is taken from a cadaver, but the disadvantages associated with this include scarcity of suitable tissue, the risk of the recipient contracting a disease from the donor, or tissue rejection [8, 9]. Prosthetic replacements (synthetic grafts) have previously been used, but they are inadequate due to wear and degeneration [10]. Surgical reconstruction techniques have limitations and do not always provide completely satisfactory long-term results in a high proportion of patients, consequently affecting their quality of life [7, 11]. Because of this dilemma, regenerative medicine could be an option, whereby *in vitro* tissue engineering of ligaments could offer a solution to the problems associated with the current surgical methods [4, 6]. Tissue-engineered ligaments could provide better performance in the long run by improved biocompatibility, better integration into host tissue, and the ability to remodel their own extracellular matrix [12].

 Tissue engineering is a method which combines knowledge from material science, engineering, molecular biology, and medicine [12]. The basic procedure normally involves using scaffolds to act as structural supports for cell growth and maturation *in vitro*, where a chemical or mechanical stimulus may also be applied to promote the formation of a functional tissue. This concept was originally developed to repair skin, cartilage, and bone, but is now being considered as a possible option to produce a functional ACL.

 It is important to have input from some of the end users, namely, the orthopaedic surgeons, on developing tissue engineering strategies so that scientists working in this area can determine whether their research is heading in the correct direction. We wanted to ascertain whether the orthopaedic surgeons that routinely carry out ACL reconstruction would choose to use a-tissue engineered ACL for implantation if it were an option and to elucidate any reservations that they may have regarding this emerging technology. Therefore, we developed an online questionnaire to investigate the current clinical opinion on using tissue- engineered ACLs to treat their future patients.

## 2. Materials and Methods

An online questionnaire was designed to ascertain how satisfied knee surgeons were with the current autografting methods for ACL reconstructions and to gain their opinions towards using a tissue-engineered ACL for implantation as an alternative method to the current autografting procedure. We also wanted to determine whether the surgeons felt it would be advantageous to surgeon, patient, and the National Health Service (NHS) to use a tissue-engineered ACL. The following questions and responses were included as described in Table 1.

Many of the questions above required “Yes, No or Do not know” answers, while others were categorised or required the input of values. The questionnaire was peer reviewed and subsequently approved by the local research ethics committee (Project no. 09/H1204/64, approved 15/10/09). Between July and October 2010, 300 orthopaedic surgeons specialising in knee surgery and soft tissue injury in the UK were contacted by e-mail and invited to participate. From this e-mailed input request, 79 surgeons responded.

The sample of surgeons contacted for inclusion in this study were those who were members of the British Orthopaedic Association (BOA), who also belonged to one of two specialist societies, the British Association of Surgery of the Knee (BASK) and the British Orthopaedic Sports Trauma Association (BOSTA). The e-mail addresses were obtained from the BOA handbook of 2006, where the invitation and link to the survey was sent to 300 surgeons. Reminders (aimed at nonresponders) were sent at 8 days, 42 days, and again at 69 days after the first invitation to ask for their feedback. The results remained anonymous and were analysed using Microsoft Excel.

## 3. Results

The overall response rate in total was 26% (79/300): we did not receive an answer from 221 surgeons. Of those who did respond, 10% (8/79) did not perform ACL reconstruction and,therefore, could not provide feedback. Most of the surgeons who completed the survey had been an orthopaedic consultant for more than 10 years (62%, 44/71), with 15% (11/71) of the respondents having been a consultant for less than 5 years. Sixteen of 71 (23%) respondents had been an orthopaedic consultant for 5 to 10 years. From the 71 surgeons, 69% (49/71) had over 10 years of experience performing ACL reconstructions, with 23% (16/71) having 5–10 years of experience, and 8% (6/71) having less than 5 years of experience.

The average number of ACL reconstructive surgeries that were performed by the respondents every month varied from 1 to 15. The majority of respondents (77%, 55/71) performed between 2 and 8 ACL reconstructions a month.

**Figure 1:**
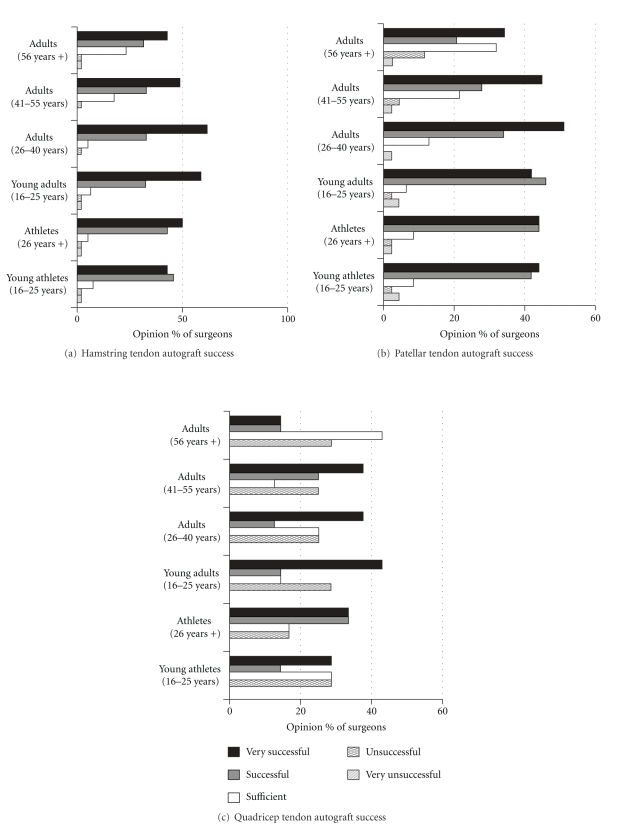
Graphs demonstrating current surgical opinion on the success rate of the use of (a) hamstring tendon, (b) patellar tendon, and (c) quadriceps tendon for the repair of anterior cruciate ligament injuries.

Forty-eight surgeons (68%, 48/71) responded to question 5 regarding their opinion of the current success of using the patellar tendon as an ACL repair. More surgeons (a total of 66/71, 93%) completed question six regarding their opinion of the treatment success of the hamstring tendon for ACL repair, whereas fewer surgeons (13%, 9/71) responded to question 7 regarding their opinion on the use of the quadriceps tendon for ACL repair.  Figure 1 shows the opinion of the respondents with regards to the success of each of these three currently used ACL treatment methods. 

The majority of surgeons (86%, 41/48) found patellar tendon autografts to be very successful/successful for treating athletes (any age) and adults of 16–40 years in age. On average, 54% (26/48) of surgeons felt that the patellar tendon was very successful/successful in patients with a lower typical daily exercise regime who were 56 years or older. Surgeons felt that the patellar tendon was either satisfactory (32%, 15/48) or very unsuccessful/unsuccessful (13%, 6/48) for these older patients.

The hamstring tendon procedure was viewed by 88% (58/66) of surgeons to be very successful/successful for athletes (any age) and patients with “normal” activity up to the age of 40. 73% (48/66) of surgeons felt that this procedure was very successful/successful for patients with “normal” activity over the age of 56. Surgeons felt that for these patients (older than 56 years of age) the hamstring tendon procedure was either sufficient (23%, 15/66) or very unsuccessful/unsuccessful (3%, 2/66).

Fewer surgeons felt that the use of quadriceps tendon was very successful/successful for young athletes (42%, 4/9) and athletes over 26 years (66%, 6/9) in comparison to the patellar tendon and hamstring tendon. In these cases, 25–28% of surgeons felt that this technique was unsuccessful for all people (all ages and activity levels).

Of 71 surgeons, 67 (94%) were familiar with the term tissue engineering and its implications as a future therapy. 86% of the surgeons (61/71) felt that they would consider using a tissue-engineered ACL as a treatment provided that it had demonstrated biological and mechanical success *in vitro *and *in vivo*. 6% (5/79) surgeons would not consider this treatment option, and a further 8% (6/79) were unsure.

The time that the surgeons stated they would be prepared to wait for a tissue-engineered ACL to be prepared varied from 2 to 26 weeks. From the 64 who answered this question, the majority (59/64) would wait up to 12 weeks. Of those 59 surgeons, 8% (5/59) felt that they would be prepared to wait 4 weeks, 44% (26/59) 6 weeks, 12% (7/59) 8 weeks, and 36% (21/59) said they would wait 12 weeks for such an implant to be ready for clinical use.

The surgical opinion of concern regarding the successful integration of a tissue-engineered ligament into the native bone was as follows: very concerned (25%, 18/71), concerned (42%, 30/71), slightly concerned (15%, 11/71), not particularly concerned (15%, 11/71), and not at all concerned (1%, 1/71).

75% (53/71) of the participating surgeons felt that it was more appropriate to use a tissue-engineered ACL which had the capability to be an exact match to the native ACL than the currently used tendon autografts. 10% (7/71) of surgeons felt that it was not more appropriate, and 14% (10/71) of surgeons did not know.

The length of time taken for the surgeons to operate using their current tendon autograft technique varied from approximately 60 to 90 minutes per operation. The surgeons stated that recovery time for these currently used autograft techniques for ACL treatment varied from 1 to 18 months, with 10% (7/71) surgeons stating 2 months, 43% (31/71) surgeons stating 6 months, and 13% (9/71) surgeons stating 9 months are needed for full recovery time after surgery. The minority (34%) suggested that a longer period was necessary.

The surgeons felt that 60% (43/71) patients would prefer to wait for a tissue-engineered ACL to be prepared for their ACL repair instead of using current techniques. Another 28% (20/71) of surgeons were unsure of what the patients response would be, and 11% (8/71) felt that the patient would probably not prefer to wait for a tissue-engineered ligament to be produced for them.

**Figure 2:**
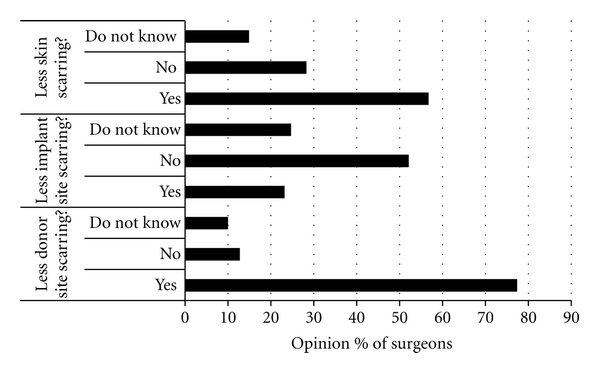
Graph to demonstrate the % of surgeons who believe that using a tissue-engineered ligament would produce less scarring in relation to location in the body.

Figure 2 shows the opinions of the surgeons regarding whether scarring will or will not be reduced as a result of using tissue-engineering as a new therapy for ACL reconstruction. 77% (55/71) of the surgeons felt there would be less scarring at the donor site as a result of using tissue-engineered implants. 62% (44/71) of surgeons believed that using a tissue-engineered ACL would reduce operative time, 24% (17/71) felt that time would not be reduced in surgery, and 14% (10/71) stated that they were unsure if surgery time would be reduced. The surgeons felt that anywhere between 5 and 30 minutes reduction on surgery time would be of significant improvement to them, with 39% (28/71) of surgeons stating that 10 to 15 minutes would make a difference to them and 21% (15/71) stating that 20 minutes would be beneficial.

When surgeons were asked whether they believed that using a tissue-engineered ligament would give patients a shorter full recovery time, 23% (16/71) said yes, 47% said no (33/71), and 30% (21/71) were unsure. When questioned further to discover how much the patient recovery time needed to be reduced for them to consider it to be a significant improvement, the surgeons replies (from a total of 47 who responded to this question) varied between 4 and 26 weeks. The majority (45/47) indicated there needed to be a reduction of up to 12 weeks. 33% (15/45) of surgeons felt that a 4-week reduction in recovery time would be significant, 38% (17/45) felt 6 weeks would, and 29% (13/45) felt that 12 weeks would be of significance. 

38% (27/71) surgeons believed that using a tissue-engineered ligament would give recovering patients reduced pain or recurring injuries, with 35% (25/71) of surgeons being unsure whether this was true. 27% (19/71) surgeons felt that a tissue-engineered ACL would not give these added benefits.

When surgeons were asked whether they would consider using a tissue-engineered construct that costs more than their current procedure (for instance, up to twice the amount) but significantly improved the patients satisfaction (resolution of instability/mobility/strength) 63% (45/71) stated yes, 10% (7/71) said no, and 27% (19/71) were unsure.

There were a variety of comments left by the surgeons when prompted. A recurring theme that surgeons were concerned about with regards to the use of a tissue-engineered ACL was of fixation. For example, how will the new device be fixed into the bone and how successfully will it integrate? Another recurring theme was the importance of reducing donor site morbidity, which appeared to be very appealing to the surgeons. Other comments included the need for initial mechanical integrity of the construct to be immediately load bearing. Many surgeons also felt that the engineered ACL should come prepared with whip stitches ready for implantation. Individual comments include the following: “Would be very useful in revision ACL reconstruction,” “The whole issue is stability. If the tissue is as good and not significantly more expensive I would use it,” “The key will be getting the graft to incorporate and pick up a blood supply. The graft will not have a nerve supply and postoperative rehab will be unchanged as I see it while patients restore proprioception. Engineered grafts may be of use in revision reconstructions and in multiligament knee injuries where autograft harvesting results in significant donor site morbidity and takes time.”

## 4. Discussion

Tissue engineering has been of increasing interest as a new and emerging therapeutic strategy. These techniques have been applied clinically with varying success for skin and cartilage. Researchers are beginning to investigate new approaches to engineer replacements for the ACL [13, 14]. It is important to include a dialogue with the end users of these products during their development to ensure the correct criteria are being addressed.

As such, this survey was targeted at surgeons who performed ACL reconstruction to gain their views on how satisfied they are with the current methods for ACL reconstruction, whether there is a need for an alternative method, and how they would feel about using a tissue-engineered ACL if it were an option. As with most surveys, there were two potential sources for bias. The first was an incomplete sampling frame because not all specialist surgeons in the UK could be contacted (unavailable/invalid addresses), and the second was represented by nonresponders. However, the data obtained are the first, to our knowledge, to give an indication of current clinical opinion of the potential of tissue engineering for the ACL. This feedback is essential to tissue engineering scientists to ensure that the correct product goal posts are set with respect to functionality and culture growth rate *in vitro*. An assessment of the potential use of this new and emerging strategy from the perspective of the end users of a tissue-engineered product is important to be incorporated from the early stages of product development.

Our data demonstrated that most of the participants had performed surgery for more than 10 years, performing from 1 to 15 reconstructions per month, and therefore regarded as experienced, making their opinions valuable to us. Generally the results implied that patellar tendon and hamstring grafts were very successful for all age groups (including athletes,) regarding their return to physical activities with no recurring injuries, whereas quadriceps grafts were very successful in patients aged <56 years, with a very high percentage rating them as “unsuccessful” compared to the patellar tendon or hamstring. The patellar tendon is known to be a very stiff, strong tissue, if not stiffer than the ACL, and is also associated with a short healing period [15], making it suitable choice for reconstructions, as confirmed by the high success rate indicated by the responses. Currently, the most commonly used grafts are patellar tendon and hamstring, but the quadriceps tendon is also becoming a popular graft, producing fewer donor site problems than harvesting of the patellar tendon, with excellent mechanical characteristics [15]. From the data obtained, the percentage of respondents who found a quadriceps graft to be very successful was much lower than for patellar tendon and hamstring, with a relatively large proportion suggesting that it was unsuccessful.

Although the current surgical procedures are successful in a high proportion of patients, surgeons would consider using alternative methods if they were an improvement over existing techniques. A high proportion would be prepared to use a tissue-engineered ACL if it were shown to have biological and mechanical success (*in vivo* and *in vitro*) and significantly improved patients' satisfaction, but there were some concerns about its successful integration into bone. It was also recognised that there would be a reduction in scarring when tissue harvesting was not required (no donor site), which would be another advantage to the patient. In particular, it may be worthwhile from a tissue engineer point of view to develop tissue-engineered ACL constructs for the older patient (56 years plus), as surgeons have less confidence in ACL reconstruction in this age group [16].

If the procedure time could be reduced using a tissue-engineered ACL, it could be of benefit to both patient and the NHS, reducing anaesthetic time and surgery costs, where a 10–30 min reduction would be needed to consider it an improvement over existing methods. Currently, the surgeons feel that tissue engineering an ACL will not significantly reduce recovery time. As such, it is important for scientists to design appropriate experiments to effectively demonstrate that the products being developed reduce recovery time. This should be translated into the appropriate animal model—for example, a reduction of an 18-month recovery time by 3 months (96% of surgeons stated that 12 weeks reduction in recovery time would be significant to them) would produce a 20% recovery time reduction. If a mouse ligament model was to normally take 10 weeks to fully recover, the tissue-engineered counterpart should demonstrate full recovery by at least 8 weeks. Scarring did not appear to be an issue for the surgeons.

Surgery time to reconstruct the ACL was typically stated to last between 60 and 90 minutes. A significant reduction of this surgery time to the surgeons would be 20 minutes. Again, this translates to an approximate reduction of surgery time of 25%. These convincing statistics need to be incorporated and considered into the preclinical animal models used by tissue engineers when testing their products. The need for ways to reduce surgery time was highlighted further when the respondents were also asked for their comments which included comments such the need for preprepared whip stitches and easy insertion techniques.

One important factor to consider is how long the *in vitro* culture can be in order to obtain the functional ligament. This survey identifies a clinical need for a stable, usable construct by 4–6 weeks. Approximately two-thirds of the surgeons surveyed were concerned about the method of integration of the tissue-engineered ACL into the host bone. These factors should be fully considered when designing such a construct, and efforts to demonstrate this efficacy should be included. Cost was not as much as a concern to the clinicians than the actual performance of the replacement ACL. The stability and potential reduction of full recovery time of the replacement ACL was far more important than the initial expense of the tissue engineered ACL. Recent reviews indicate the need for improving ligament repair [17, 18] as such, this current study indicates that clinicians may be open to improving current methods using tissue-engineering strategies.

## 5. Conclusions

The majority of British surgeons were familiar with tissue engineering as a concept and are very open to its potential use. Current treatments for patients over 56 years of age have a greater need for improvement than for younger patients. Greater patient awareness is needed with regards to tissue engineering as a potential therapy for an increase in potential acceptance of new strategies by these end users. Surgeons clearly detailed a need to have a fully load-bearing construct for implantation. Therefore, it is important for tissue engineers to ensure that they have reliable mechanical integrity data of their developing constructs. Currently, surgeons are not convinced that tissue-engineered ligaments could shorten the recovery time of the ACL in comparison to the current methods used. Again, it is important that the data gathered by the tissue engineer researchers effectively demonstrate this potential. Improvements to currently used procedures need to reduce surgery time by at least 20 minutes and reduce the patient time to full recovery by a minimum of 12 weeks before surgeons feel that there is a benefit. If these criteria are met, it was not deemed as a problem if the operation costs increased up to double the current costs. Lastly, there is a need to have these tissue-engineered constructs ready for use after a minimum of 4–6 weeks *in vitro* culture. Any potential longer *in vitro* culture time becomes unattractive for the surgeons.

## Figures and Tables

**Table 1:** Questionnaire used online for orthopaedic consultant feedback.

Question	
1	How many years have you been a consultant?

2	Do you perform anterior cruciate ligament (ACL) reconstructions?

3	Approximately how many ACL reconstructions do you perform each month?

4	How long have you been performing ACL reconstructions?

5	How successful do you rate patellar tendon ACL reconstructions? (e.g., the patient being able to return to physical activities without experiencing another injury to the reconstructed ACL)

6	How successful do you rate hamstring ACL reconstructions? (e.g., the patient being able to return to physical activities without experiencing another injury to the reconstructed ACL)

7	How successful do you rate quadriceps ACL reconstructions? (e.g., the patient being able to return to physical activities without experiencing another injury to the reconstructed ACL)

8	Are you familiar with tissue engineering as future clinic therapy? (Definition of tissue engineering: To grow autologous tissue *in vitro* in order to replace damaged body parts.)

9	If tissue engineering an ACL for the patient were an option (either through the NHS or privately), would you consider using a newly developed tissue-engineered ACL? (If it had shown mechanical and biological success *in vitro and in vivo*)

10	If an autologous tissue were tissue-engineered in the laboratory, what time limit would you see as acceptable from the moment the patients cells were harvested to the moment the engineered ACL was ready for implantation?

11	If you were to hypothetically use a tissue-engineered ACL, would you be concerned about the successful integration of the engineered ACL into the bone?

12	An engineered ACL could be an exact match to the native ACL. Do you feel that this would be more appropriate for implantation than a hamstring, quadriceps, or patellar tendon (which are only similar in tissue type to the ACL and not an exact match)?

13	Approximately how long on average does your current treatment strategy for ACL replacement take (a) regarding operation length (b) regarding full recovery time with no pain

14	Do you think it is likely that some patients would prefer to wait to receive a tissue-engineered ACL from their own cells, rather than receiving the current surgical ACL reconstruction using their own patellar tendon/hamstring tendon/quadriceps tendon?

15	With respect to tissue engineering
	(a) Do you believe that using a tissue-engineered ligament would produce less scarring? Donor site scarring (e.g. patellar tendon, hamstring, quadricep), implant site scarring, skin scarring as a result from donor tissue harvest?
	(b) Do you believe that using a tissue-engineered ligament would take less surgical time?
	(c) By how much would surgical time need to be reduced for you to consider it to be a significant improvement?
	(d) Do you believe that using a tissue-engineered ligament would give patients a shorter full recovery time?
	(e) By how much would recovery time need to be reduced for you to consider it to be a significant improvement?
	(f) Do you believe that using a tissue-engineered ligament would give recovering patients reduced pain or recurring injuries?

16	Currently ACL reconstructions cost *£*2,061 (NHS) and *£*3,500-*£*5,000 (privately). If a tissue-engineered construct cost more than your current procedure (for instance, up to twice the amount) but significantly improved the patient's satisfaction (resolution of instability/mobility/strength), would you consider using this technique?

17	Any other personal suggestions? For example, what do you see as an advantage/disadvantage regarding using tissue-engineered constructs? Do you see a need to improve current surgical techniques?
